# Contribution of vascular endothelial growth factor to the Nottingham prognostic index in node-negative breast cancer

**DOI:** 10.1054/bjoc.2001.2019

**Published:** 2001-09

**Authors:** D Coradini, P Boracchi, M Grazia Daidone, C Pellizzaro, P Miodini, M Ammatuna, G Tomasic, E Biganzoli

**Affiliations:** Unità Operativa Determinanti Biomolecolari nella Prognosi e Terapia, Unità di Statistica Medica e Biometria, Unità di Anestesia e Rianimazione, Unità di Anatomia Patologica, Istituto Nazionale per lo Studio e la Cura dei Tumori, Istituto di Statistica Medica e Biometria, Università degli Studi di Milano, Milan, Italy

**Keywords:** flexible modelling, N-negative primary breast cancer, NPI, VEGF

## Abstract

The prognostic contribution of intratumour VEGF, the most important factor in tumour-induced angiogenesis, to NPI was evaluated by using flexible modelling in a series of 226 N-primary breast cancer patients in which steroid receptors and cell proliferation were also accounted for. VEGF provided an additional prognostic contribution to NPI mainly within ER-poor tumours. © 2001 Cancer Research Campaignhttp://www.bjcancer.com


British Journal of Cancer (2001) **85**(6), 795–797
© 2001 Cancer Research Campaign
doi: 10.1054/ bjoc.2001.2019, available online at http://www.idealibrary.com on IDEAL

http://www.bjcancer.com

### Contribution of vascular endothelial growth factor to the Nottingham prognostic index in node-negative breast cancer

**D Coradini[1], P Boracchi[5], M Grazia Daidone[1], C Pellizzaro[1], P Miodini[1], M Ammatuna[3], G Tomasic[4] and E Biganzoli[2]**

[1]Unità Operativa Determinanti Biomolecolari nella Prognosi e Terapia; [2]Unità di Statistica Medica e Biometria; [3]Unità di Anestesia e Rianimazione; [4]Unità di Anatomia Patologica, Istituto Nazionale per lo Studio e la Cura dei Tumori; [5]Istituto di Statistica Medica e Biometria, Università degli Studi di Milano, Milan, Italy

**Summary** The prognostic contribution of intratumour VEGF, the most important factor in tumour-induced angiogenesis, to NPI was evaluated by using flexible modelling in a series of 226 N-primary breast cancer patients in which steroid receptors and cell proliferation were also accounted for. VEGF provided an additional prognostic contribution to NPI mainly within ER-poor tumours. © 2001 Cancer Research Campaign http://www.bjcancer.com

**Keywords**: flexible modelling; N-negative primary breast cancer; NPI; VEGF

Despite substantial progress in early detection and treatment, breast cancer remains the second commonest malignancy in the female population. It affects approximately 1 in 10 women and is responsible for 23.7% of all cancer deaths (Breast Cancer Statistics, 2000). Comprehensive research has been conducted to identify clinically useful prognostic factors in primary breast cancer, with special efforts to identify among node-negative (N–) patients those with an aggressive phenotype who need adjuvant systemic treatments and those with indolent tumours who are likely to be cured by local–regional therapy. A unique, highly discriminating prognostic factor has not yet been proposed and, in consideration of the complex biology of the neoplasm, it is unlikely that one will be identified, although novel promising prognosticators are emerging. Moreover, sufficient and univocal evidence has not yet been provided for assessing either the prognostic strength of biological markers or their actual clinical impact. In fact, a few studies have evaluated the adjunctive contribution of novel factors to already validated scores proposed for breast cancer and based on different morphopathobiologic features of proven prognostic utility (Altman and Lyman, 1998; Goldhirsch et al, 1998; Hayes et al, 1998).

The Nottingham prognostic index (NPI) can be considered a gold standard for prognosis since it is based on morphopathologic features such as lymph node stage, tumour size and histologic grading of malignancy. It has been prospectively validated in several studies and has proved to maintain over time a valuable discriminating power in differentiating patients in low-, intermediate- and high-risk subsets (Haybittle et al, 1982; Galea et al, 1992; Balslev et al, 1994). Although lymph node stage has a pivotal role in the NPI definition, NPI is a validated prognostic discriminator also in N– patients (Saurbrei et al, 1997) and can be considered as a baseline reference for the evaluation of the additional prognostic role of biological variables.

Experimental studies have suggested tumour-induced neoangiogenesis as an important step in the evolution of malignant tumours (Ellis and Fidler, 1996; Yosiji et al, 1996). Recent clinical evidence indicates that its evaluation, either as microvessel count or as vascular endothelial growth factor (VEGF) content, significantly contributes to identify patients with a worse prognosis, mainly within the N– subset (Gasparini, 1996; Heimann et al, 1996) but also within endocrine adjuvant setting (Linderholm et al, 2000). Since steroid receptor content is currently used in clinical practice and tumour proliferative activity is a recognized prognostic factor in N– patients (Hayes et al, 1998), the aim of the present study was to evaluate the adjunctive prognostic contribution of intratumour VEGF concentration to a widely adopted prognostic classification (NPI), taking into account the effect of steroid receptors and cell proliferation. For evaluation of the prognostic impact of VEGF concentration in the continuous scale of measurement, flexible regression and bootstrap techniques have been adopted.

### MATERIALS AND METHODS

The present study was carried out on a series of 226 N– primary breast cancer patients subjected, between May 1991 and May 1993, only to radical or conservative surgery plus radiotherapy and to complete axillary dissection until relapse and followed up for a median of 75 months (range, 4–98) at the Istituto Nazionale Tumori of Milan. Information on the cases was also available for steroid receptors, evaluated by the dextran-coated charcoal technique (Ronchi et al, 1986), and tumour proliferative activity, expressed as the thymidine labelling index (TLI) (Silvestrini et al, 1995). The NPI was determined according to Elston and Ellis (1991).

VEGF expression was measured by a quantitative enzyme immunoassay technique. The role of NPI, VEGF, oestrogen and progesterone (ER, PgR) content, and TLI on disease-free survival

*Received 2 February 2001*
*Revised 27 June 2001*
*Accepted 4 July 2001*

*Correspondence to:* Danila Coradini

(DFS, defined as time from surgery to local–regional recurrence, distant metastasis or contralateral tumours; 63 events) was investigated by the Cox model, once the proportional hazard assumption was verified (Grambsch and Therneau, 1994). On the basis of prior knowledge, only linear terms were considered for ER, PgR and TLI after logarithmic transformation (Silvestrini et al, 1996). For VEGF, a restricted cubic spline transformation (Durrleman and Simon, 1989) was initially considered on its logarithmic values, whereas only the linear term was finally adopted. Among pre-specified interactions (VEGF × TLI, VEGF × NPI, VEGF × ER) only VEGF × ER was selected. The additional prognostic contribution of biological variables was also evaluated by a bootstrap re-sampling technique (200 samples) applied to a backward variable selection, starting from a model including all variables and the interaction. Model comparisons were performed on the basis of Akaike Information Criterion (AIC) (Collett, 1994), considering 3 different penalty factors for the degrees of freedom (i.e. 2, 3, 4) – the first corresponding to that traditionally used for AIC and the other 2 allowing for a more conservative selection. Model predictive ability was measured by Harrell's c statistic (Harrell et al, 1996). To illustrate the effect of covariates, the expected survival curves, calculated from Cox model results, were plotted according to fixed covariate values. Selected values were the 25th, 50th or 75th percentiles for VEGF, the 25th or 75th percentiles for ER, low (1) and intermediate (2) NPI. TLI and PgR were fixed to the median values of their distributions.

### RESULTS AND DISCUSSION

The values of VEGF ranged from 0 to 337.3 with 30.7, 57.4, 97.4, as 25th, 50th, 75th percentiles, respectively. The DFS curves according to NPI levels are shown in Figure 1A: since only N– patients were considered, the NPI classification is only a 2-level score: low and intermediate. The difference between DFS curves was statistically significant (hazard ratio (HR) for replase: intermediate NPI vs low NPI, 1.71; 95% confidence limits (CL), 1.03–2.83; $P = 0.039$). In the multivariate regression model (Table 1), using the conventional penalty factor of 2 for AIC, TLI was retained in the model in 67.5%, ER in 71.5%, VEGF in 94% and VEGF*ER interaction in 66.5% of the bootstrap samples. Moreover, VEGF was retained in 90.5% and 76.6% of the bootstrap samples with the increased penalty terms.

The predictive ability of the model was c = 0.655, whereas considering NPI only and NPI plus steroid receptors and TLI, the c statistic dropped to 0.571 and 0.623, respectively. To provide a graphic display of model results, expected DFS curves were drawn for selected combinations of variable values (Figure 1B, ER-poor tumours; Figure 1C, ER-rich tumours). The present findings seems to suggest a discriminatory effect of VEGF in identifying patients who differ in prognosis in NPI = 1 and NPI = 2 groups (5-year probability of relapse for low vs high VEGF: 12% vs 20% and 19% vs 31%, respectively) mainly in ER-poor tumours (Figure 1B). For these patients, the estimated HR for disease for high vs low VEGF was 1.79 (95% CL, 1.15–2.79). Conversely, in ER-rich tumours, i.e., in tumours with an ER content greater than 126 fmol mg$^{-1}$ cytosolic protein) (Figure 1C), VEGF was unable to segregate patients with different prognosis, and the HR was 1.18 (95% CL, 0.78–1.79). In ER-poor tumours angiogenesis might be at an initial stage and thus the presence of high VEGF levels could indicate the activation of such a process. Conversely, in the ER-rich subset a functional hormone control seems able to counteract angiogenesis activation in both NPI groups and, as suggested by a previous study (Coradini et al, 2000), a longer follow-up could be

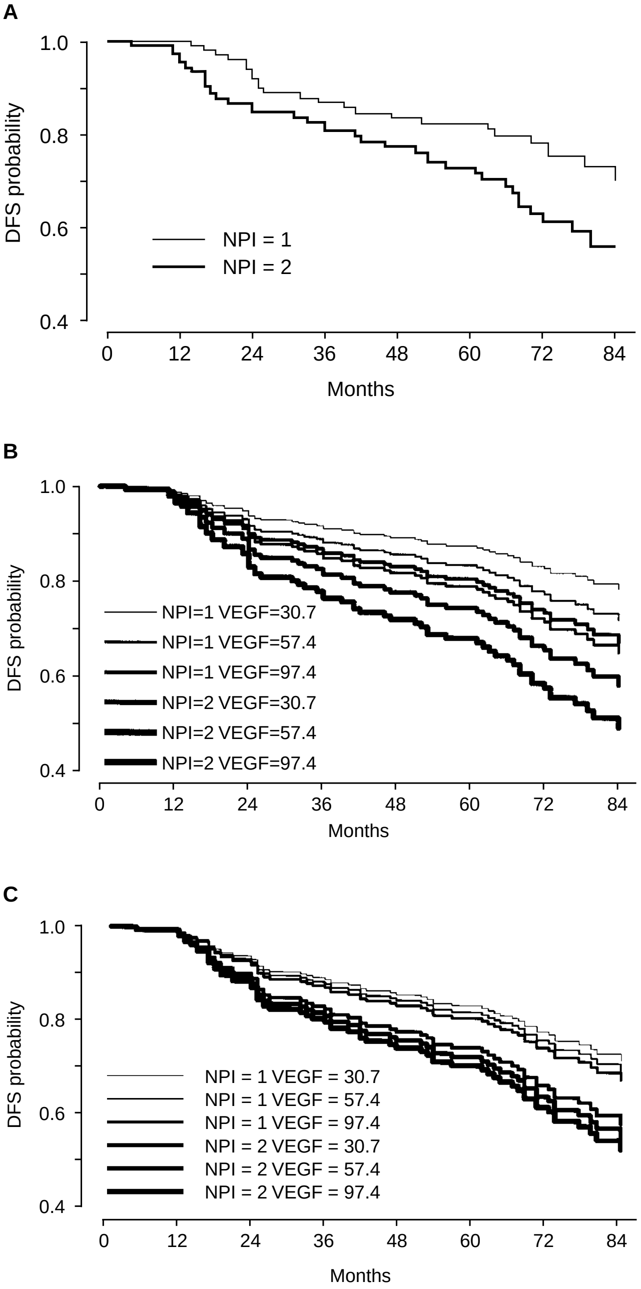

**Figure 1** Disease-free survival curves for 226 patients with node-negative resectable breast cancer. (**A**) Kaplan–Meier curves according to NPI. (**B**) Expected disease-free survival curves calculated from the Cox multiple regression model as a function of NPI (1,2) and VEGF (30.7, 57.4, 97.4 pg mg$^{-1}$ cytosolic protein, corresponding to the 25th, 50th and 75th percentiles of the distribution) for patients with ER-poor tumours (17 fmol mg$^{-1}$ cytosolic protein, corresponding to the 25th percentile of the distribution). TLI and PgR are fixed to the median values of their distributions: 2.8% and 68 fmol mg$^{-1}$ cytosolic protein, respectively. VEGF expression was measured by a quantitative enzyme immunoassay technique following the manifacturer's instruction (CYTimmune Science). Each sample was run in duplicate and calibration was performed according to the method proposed by O'Connell et al (1993). (**C**) Expected disease-free survival curves from the Cox multiple regression model as a function of NPI and VEGF (30.7, 57.4, 97.4 pg mg$^{-1}$ cytosolic protein, corresponding to the 25th, 50th and 75th percentiles of the distribution) for patients with ER-rich tumours (126 fmol mg$^{-1}$ cytosolic protein, corresponding to the 75th percentile of the distribution). TLI and PgR are fixed to the median values of their distributions: 2.8% and 68 fmol mg$^{-1}$ cytosol protein, respectively

© 2001 Cancer Research Campaign

**Table 1** Multivariate analysis for disease-free survival

| Model terms | b (SE)[a] | Model effects | DF[b] | $\chi^{2\,c}$ | *P* |
|---|---|---|---|---|---|
| | | Full model | 6 | 14.44 | 0.0140 |
| NPI | 0.498 (0.277) | Factor | 1 | 3.25 | 0.0715 |
| ER[d] | 0.788 (0.452) | Factor + Interaction | 2 | 3.40 | 0.1823 |
| PgR[d] | 0.025 (0.067) | Factor | 1 | 0.14 | 0.7053 |
| TLI[d] | 0.407 (0.242) | Factor | 1 | 2.84 | 0.0918 |
| VEGF[d] | 1.031 (0.430) | Factor + Interaction | 2 | 6.76 | 0.0340 |
| VEGF[d]*ER[d] | –0.183 (0.100) | Interaction | 1 | 3.32 | 0.0686 |

[a]Estimated regression coefficient. In parenthesis, standard error.
[b]Degree of freedom.
[c]Wald statistics for model effects.
[d]Logarithmic transformation.

necessary to observe the disruption of the hormone-related growth control and angiogenesis balance.

### ACKNOWLEDGEMENTS

Supported in part by the Associazione Italiana per la Ricerca sul Cancro (AIRC). We thank Prof Ettore Marubini for his valuable comments.

### REFERENCES

Altman DG and Lyman GH (1998) Methodological challenges in the evaluation of prognostic factors in breast cancer. *Breast Cancer Res Treat* **52**: 289–303

Balslev I, Axelsson CK, Zedeler K, Rasmusen BB, Carstensen B and Mouridsen HT (1994) The Nottingham Prognostic Index applied to 9149 patients from the studies of the Danish breast cancer cooperative group (DBCG). *Breast Cancer Res Treat* **32**: 281–290

Breast Cancer Statistics (2000) *J Natl Cancer Inst* **92**: 445

Collett K (1994) *Modelling survival data in medical research*. Chapman & Hall: London

Coradini D, Daidone MG, Boracchi P, Biganzoli E, Oriana S, Bresciani G, Pellizzaro C, Tomasic G, Di Fronzo G and Marubini E (2000) Time-dependent relevance of steroid receptors in breast cancer. *J Clin Oncol* **18**: 2702–2709

Durrleman S and Simon R (1989) Flexible regression models with cubic splines. *Stat Med* **8**: 551–561

Ellis LM and Fidler IJ (1996) Angiogenesis and metastasis. *Eur J Cancer* **32A**: 2451–2460

Elston CW and Ellis IO (1991) Pathological prognostic factor in breast cancer. The value of histological grade in breast cancer: experience from a large study with long-term follow-up. *Histopathol* **19**: 403–410

Galea MH, Blamey RW, Elston CE and Ellis IO (1992) The Nottingham Prognostic Index in primary breast cancer. *Breast Cancer Res Treat* **22**: 207–219

Gasparini G (1996) Clinical significance of the determination of angiogenesis in human breast cancer: update of the biological background and overview of the Vicenza Studies. *Eur J Cancer* **32A**: 2485–2493

Goldhirsch A, Glick JH, Gelber RD and Senn H-J (1998) Meeting highlights: International consensus panel on the treatment of primary breast cancer. *J Natl Cancer Inst* **90**: 1601–1608

Grambsch P and Therneau T (1994) Proportional hazards tests and diagnostics based on weighted residuals. *Biometrika* **81**: 515–526

Harrell FE, Lee KL and Mark DB (1996) Tutorial in biostatistics. Multivariable prognostic models: issue in developing models, evaluating assumptions and adequacy, and measuring and reducing errors. *Stat Med* **15**: 361–387

Haybittle JL, Blamey RW, Elson CW, Johnson J, Doyle PJ, Campbell FC, Nicholson RI and Griffiths K (1982) A prognostic index in primary breast cancer. *Br J Cancer* **45**: 361–366

Hayes DF, Trock B and Harris AL (1998) Assessing the clinical impact of prognostic factors: when is “statistically significant” clinically useful? *Breast Cancer Res Treat* **52**: 305–319

Heimann R, Ferguson D, Powers C, Recant WM, Weichselbaum RR and Halmann S (1996) Angiogenesis as predictor of long-term survival for patients with node-negative breast cancer. *J Natl Cancer Inst* **88**: 1764–1769

Linderholm B, Grankvist K, Wilking N, Johansson M, Tavelin B and Henriksson R (2000) Correlation of vascular endothelial growth factor content with recurrences, survival, and first relapse site in primary node-positive breast carcinoma after adjuvant treatment. *J Clin Oncol* **18**: 1423–1431

O’Connell MA, Belanger BA and Haaland PD (1993) Calibration and assay development using the four parameter logistic model. *Chemometrics and Intelligent Laboratory Systems* **20**: 97–114

Ronchi E, Granata G, Brivio M, Coradini D, Miodini P and Di Fronzo G (1986) A double-labeling assay for simultaneous estimation and characterization of estrogen and progesterone receptors using radioiodinated estradiol and tritiated Org 2058. *Tumori* **72**: 251–257

Saurbrei W, Hubner K, Schmoor C and Schumacher M (1997) Validation of existing and development of new prognostic classification schemes in node-negative breast cancer: German breast cancer study group. *Breast Cancer Res Treat* **42**: 149–163

Silvestrini R, Daidone MG, Luisi A, Boracchi P, Mezzetti M, Di Fronzo G, Andreola S, Salvadori B and Veronesi U (1995) Biologic and clinicopathologic factors as indicators of specific relapse types in node-negative breast cancer. *J Clin Oncol* **13**: 697–704

Silvestrini R, Daidone MG, Benini E, Faranda A, Tomasic G, Boracchi P, Salvadori B and Veronesi U (1996) Validation of p53 accumulation as a predictor of distant metastasis at 10 years of follow-up in 1400 node-negative breast cancers. *Clin Cancer Res* **2**: 2007–2013

Yoshiji H, Gomez DE, Shibuya M and Thorgeirsson UP (1996) Expression of vascular endothelial growth factor its receptor and other angiogenic factors in human breast cancer. *Cancer Res* **56**: 2013–2016

*© 2001 Cancer Research Campaign* 
